# Defining the levels of the chromatin landscape using the I-CATCH system

**DOI:** 10.1038/s41598-026-65001-w

**Published:** 2026-08-06

**Authors:** Bradley M. Downs, Jiumei Hu, Joon Soo Park, Hanran Lei, Tra My Hoang, Thomas R. Pisanic, Tza-Huei Wang

**Affiliations:** 1 Institute for NanoBioTechnology, Johns Hopkins University, Baltimore, MD 21218 USA; 2 Department of Mechanical Engineering, Johns Hopkins University, Baltimore, MD 21218 USA; 3 Department of Chemistry, Konkuk University, Seoul, 05029 South Korea; 4 Department of Biomedical Engineering, Johns Hopkins University, Baltimore, MD 21218 USA; 5 MilliporeSigma, 9820 Darnestown Rd, Rockville, MD 20850 USA; 6 Department of Oncology, The Sidney Kimmel Cancer Center, Johns Hopkins University School of Medicine, Baltimore, MD 21205 USA

**Keywords:** Biological techniques, Cell biology, Computational biology and bioinformatics, Genetics, Molecular biology

## Abstract

Alterations of the epigenome, including histone tail post-translational modifications (PTMs), have been shown to regulate the expression of genes important for driving multiple cellular processes including cellular differentiation, aging and tumorigenesis. Although the ability to measure histone tail PTM levels is integral to the understanding of chromatin biology, because of technological limitations, the proportion of nucleosomes that have histone tail PTMs at genomic loci remain undefined. To overcome this limitation, we developed the *i*mmuno-profiling of d*Ca*s9 *t*argeted *ch*romatin (I-CATCH) system, a technology designed to measure and normalize the levels of nucleosomes and co-occurring histone tail PTMs at targeted loci. Here, we show that the I-CATCH system has high analytical performance for measuring the levels of histone tail PTMs and we report the normalized levels of multiple histone tail PTMs at different genomic loci from both HeLa cells and human plasma. While further studies are required to determine the full utility of the I-CATCH system, we are hopeful that the I-CATCH system will help enable the ability to investigate new important features of chromatin biology.

**Supplementary Information:**

The online version contains supplementary material available at https://doi.org/10.1038/s41598-026-65001-w.

## Introduction

In the human genome, DNA is wrapped around nucleosomes, which are comprised of eight histone proteins and form the repeating unit of chromatin. Nucleosomes are composed of two copies of each of the core histone proteins (H2A, H2B, H3 and H4) with each histone having an N-terminal tail which can be enriched with a variety of histone tail post-translational modifications (PTMs)^1^. Studies have shown that multiple sites on the histone tails can be modified simultaneously by different chemical groups, such as acetylation, methylation, and phosphorylation, and more than 40 known histone tail PTMs and over 4,000 patterns have been measured^2–5^. It is well-established that co-occurring histone tail PTMs work in concert with each other to regulate gene expression.

Modifications of the epigenome, including histone tail PTMs, drive multiple cellular processes including cellular differentiation and aging and is recognized as one of the earliest, most frequent, and robust alterations in cancer^6–10^. Studies have shown that histone tail PTM enrichment level changes at bivalent promoters, which are genomic regions that contain both active H3K4me3 and repressive H3K27me3 histone tail PTMs, are frequently altered in tumors and have been shown to regulate the expression of genes important for stemness, proliferation and metastasis^11–16^. Although there are multiple FDA-approved cancer therapeutics designed to inhibit genes that regulate histone tail PTMs (such as EZH2 and HDAC inhibitors), due to technological limitations, it is extremely difficult to determine the proportion of nucleosomes that have histone tail PTMs at genomic loci important for tumor progression in cancer patients. Thus, the ability to efficiently administer and identify individuals that are likely to respond to these therapeutics continues to be challenging.

Technologies commonly used to determine the genomic location and enrichment levels of histone tail PTMs, such as Chromatin Immunoprecipitation Sequencing (ChIP-Seq), use antibodies to target and capture DNA-bound chromatin. After the captured genomic DNA fragments are purified, they are commonly analyzed using next-generation sequencing (NGS), which can identify protein enrichment across the genome by determining the number (depth) of NGS reads mapped to each genomic loci. Furthermore, with highly controlled experiments, it is also possible to determine whether histone tail PTM levels change.

The limitations of ChIP-based technologies arise from both the usage of antibodies to capture proteins and NGS libraries to determine the genomic locations of the DNA protein complexes^17^. Since genomic sequences lacking the histone tail PTM are discarded during the antibody capturing step, the proportion of nucleosomes with a histone tail PTM at specific loci cannot be determined simultaneously. Another limitation plaguing ChIP-Seq technology is that the nucleotides comprising the DNA sequence can affect its incorporation into the NGS library, commonly referred to as the GC bias. Because of this GC bias, NGS enrichment levels cannot be compared between different genomic loci or between individuals. Thus, the information that can be derived from the mapped NGS reads from a single experiment is limited and is generally used to determine whether the levels are statistically enriched. This binary enrichment information provides little insight into histone tail PTM biology as it is well-established that gene expression is regulated by histone tail PTM level changes. Although the biological functions of nucleosomes have been studied for over 50 years^18^, the proportion of nucleosomes with histone tail PTMs at genomic loci remain completely undefined, which has significantly limited our understanding of chromatin biology.

To overcome these limitations, we developed the first technology that can simultaneously measure and normalize the levels of co-occurring histone tail PTMs at targeted genomic loci, named the *i*mmuno-profiling of d*Ca*s9 *t*argeted *ch*romatin (I-CATCH) system. The I-CATCH system first uses the capturing ability of enzymatically dead Cas9 (dCas9) biotin-bound protein to target genomic loci and streptavidin magnetic beads to pull down nucleosome-bound DNA. Next, it uses a multiplexed immuno-qPCR assay, using antibodies for the nucleosome histone 3 core protein and histone tail PTMs H3K4me3 and H3K27me3, to measure the number of captured nucleosomes and multiple histone tail PTMs simultaneously. By using a standard curve of recombinant nucleosome controls with known quantities, the number of captured nucleosomes and multiple histone tail PTMs at specific loci can be measured and normalized.

In this study, we validated the system by first verifying that the three antibodies can bind simultaneously to a nucleosome without steric hindrance. We then showed that the multiplexed immuno-qPCR assay was highly linear across a range of both nucleosome concentrations and histone tail PTM percentages. Next, we measured the levels of multiple histone tail PTMs normalized to the number of nucleosomes of several loci of HeLa cell chromatin and found high concordance between ChIP-Seq enrichment calls and the levels of histone tail PTMs measured using the I-CATCH system. We then investigated whether the I-CATCH system could measure the levels of histone tail PTMs of cell-free nucleosomes from human plasma. We found similar histone tail PTM levels between the plasma and HeLa cells when measuring loci enriched for both H3K4me3 and H3K27me3 and at loci absent of both histone tail PTMs. Furthermore, we found that the sequential capture of different loci from the same plasma sample, a required method to measure multiple loci from a single sample, did not affect the ability of the I-CATCH system to measure the levels of multiple histone tail PTMs. Thus, we conclude that the I-CATCH system can measure and normalize the levels of multiple histone tail PTMs at targeted genomic loci. In future studies, we plan to further investigate how this approach can expand our knowledge of chromatin biology and we will continue to determine the clinical utility of the I-CATCH system.

## Results

### Overview of the I-CATCH system

**Fig. 1:**
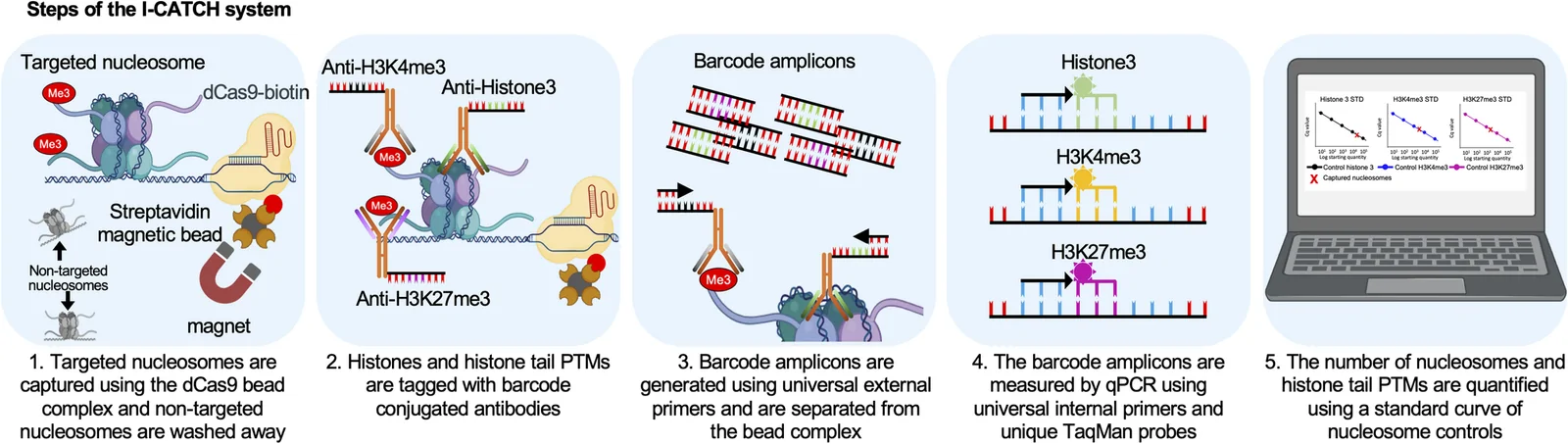
Illustration showing the multiple steps of the I-CATCH system.

Here, we introduce the I-CATCH system, a technology designed to measure and normalize the levels of nucleosomes and multiple histone tail PTMs. The I-CATCH system combines biotinylated dCas9-capturing technology with a multiplexed immuno-qPCR assay to measure the levels of multiple histone tail PTMs and histone core proteins at targeted genomic loci. In this technology, we use enzymatically dead Cas9 (dCas9), which has two amino acid changes that inhibit its ability to cleave DNA while preserving its ability to bind to targeted DNA loci through guide RNA-DNA hybridization, allowing it to bind to the nucleosome-bound genomic loci of interest^19–22^. After hybridization, we pull down the nucleosome–DNA–biotinylated dCas9 complex using streptavidin magnetic beads. Next, the captured nucleosomes and histone tail PTMs are tagged with antibodies containing DNA barcode conjugates and immuno-qPCR is used to measure the number of tags. For the immuno-qPCR assay, we chose to utilize nested qPCR technology as it is well-suited for large multiplexed qPCR measurements and it allows for the separation of amplicons and bead complexes^23,24^. In brief, nested qPCR uses a two-step PCR approach. In the first PCR, all barcode sequences are amplified using universal external primers that target the terminal ends of the barcode sequences. We then separated the bead complexes from the amplicons to prevent potential bead-fluorescence interference during the second qPCR step. In the second qPCR step, universal internal primers and TaqMan probes specific for the barcode sequences are used to measure the number of barcode amplicons. The sequences for the barcodes, primers, and probes are listed in Table 1. We then quantify the levels of the nucleosomes and histone tail PTMs by comparing the qPCR results of the targeted nucleosomes to a standard curve of known histone tail PTM quantities. An illustration of the I-CATCH system is shown in Fig. 1.

**Table 1:** sgRNA, DNA, primers and probes sequences.

Name	Primer/Sequence 5’ to 3’	Location (hg19)	Size (bp)
Barcode sequence 1	Thiol/CATCTGCCTGAGTATGAAGTTGTCGATGCTATCTCTACACCTAGTCTCAGTTCACGTCACATAACACTACAGCTACAGATACGTCCTAACTGACATCAAG		100
Barcode sequence 2	Thiol/CATCTGCCTGAGTATGAAGTTGTCGATGCTATCTCTTCCAGTGATTCCATGATGACGTCTGATACACTACAGCTACAGATACGTCCTAACTGACATCAAG		100
Barcode sequence 3	Thiol/CATCTGCCTGAGTATGAAGTTGTCGATGCTATCTCTATGCGAAGTCTGTCACCGATTTAAGCACACTACAGCTACAGATACGTCCTAACTGACATCAAG		99
External F	CATCTGCCTGAGTATGAA		
External R	CTTGATGTCAGTTAGGAC		
Internal F	GTTGTCGATGCTATCTCT		
Internal R	GTATCTGTAGCTGTAGTGT		
Barcode1 probe	FAM- ACACCTAGTCTCAGTTCACGTCACATA -MBD		
Barcode2 probe	HEX- TCCAGTGATTCCATGATGACGTCTGAT -MBD		
Barcode3 probe	TEX- ATGCGAAGTCTGTCACCGATTTAAGC -MBD		
SOX9 sgRNA	CATGAAGATGACCGACGAGCAGG	Chr17:70117552–70,117,575	
MAT2A sgRNA	CAGACGACGTCCCAAGGCATAGG	Chr2:85767016–85,767,039	
IGFBP2 sgRNA	CTGGTCTTAAGACGCCAAGCTGG	Chr2:217496909–217,496,932	
PLK5 sgRNA	GTCCACCTGGTGTCACGAATGGG	Chr19:1522886–1,522,909	

### The immuno-qPCR assay has high analytical performance for measuring recombinant nucleosome controls

**Fig. 2:**
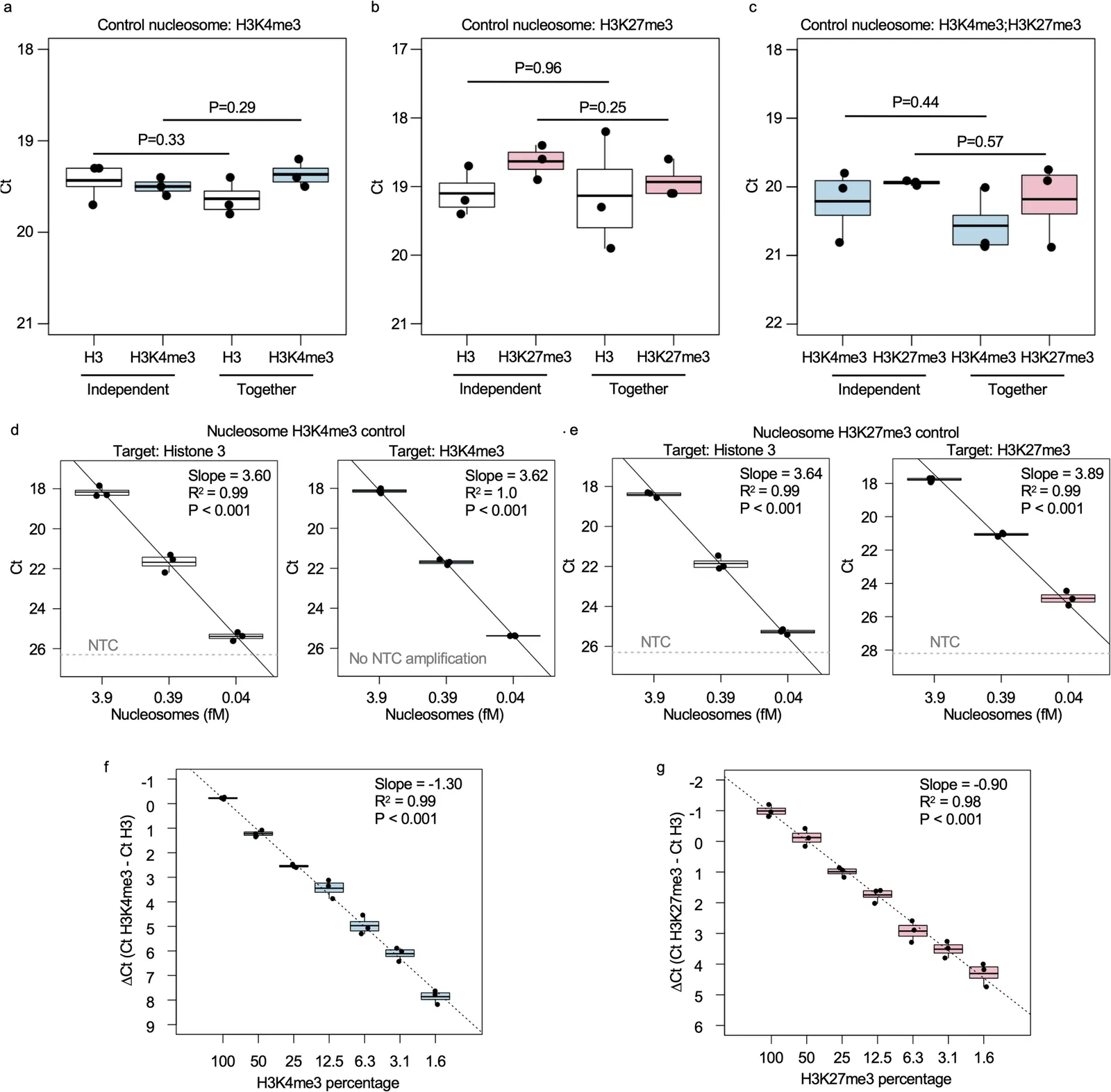
Boxplots showing the ability to use the conjugated antibodies anti-histone3, anti-H3K4me3 and anti-H3K27me3 to measure the recombinant nucleosome controls with (**a**) H3K4me3, (**b**) H3K27me3 and (**c**) both H3K4me3 and H3K27me3 histone tail PTMs independently or together. *N* = 3 for each condition; P=Student’s T-test, two-sided. Box plot showing the performance of the immuno-qPCR in measuring a range of nucleosome concentrations with (**d**) H3K4me3 and (**e**) H3K27me3 histone tail PTMs. Box plots showing the performance of the immuno-qPCR assay in measuring a range of (**f**) H3K4me3 and (**g**) H3K27me3 histone tail PTM percentages. *N* = 3 from independent aliquots of each condition; NTC = no template control Ct; P = coefficient, two-sided. Center line, mean; box limits, upper and lower quartiles; whiskers, 1.5x interquartile range; points, data.

After the barcoded sequences were conjugated to anti-histone3, anti-H3K4me3 and anti-H3K27me3 antibodies, we investigated whether there was steric hindrance when using multiple conjugated antibodies simultaneously. We found that the qPCR measurements of histone 3 and histone tail PTMs of the recombinant nucleosomes with only H3K4me3, only H3K27me3, and co-occurring H3K4me3 and H3K27me3 did not change when using the conjugated antibodies independently or together (Fig. 2a–c). Thus, we conclude that there is no steric hindrance between the anti-histone 3, anti-H3K4me3 and anti-H3K27me3 conjugated antibodies and that the I-CATCH system can measure co-occurring H3K4me3 and H3K27me3.

Next, we investigated the analytical performance of the I-CATCH system’s immuno-qPCR assay using recombinant nucleosomes with known histone tail PTMs. In a duplexed immuno-qPCR reaction, we found high degrees of linearity (R^2^ ≥ 0.99) and reproducibility (coefficient *P* < 0.001) when measuring 10-fold serial dilutions of the recombinant nucleosome controls (Fig. 2d and e). While we found that anti-H3K4me3 had no background signal in the I-CATCH system, both anti-histone 3 and anti-H3K27me3 background signal was lower than 0.04fM of the recombinant nucleosome controls (Fig. 2d and e). While this result shows that the immuno-qPCR with a standard curve of recombinant nucleosome controls has high analytical performance for quantifying the amounts of nucleosomes and histone tail PTMs, which could be useful for measuring circulating tumor DNA, we believe that normalizing the amount of histone tail PTMs to the number of nucleosomes adds important biological context when measuring histone tail PTM levels.

To investigate the performance of the I-CATCH system when measuring and normalizing the levels of multiple histone tail PTMs to the number of nucleosomes, we mixed the H3K4me3 with the H3K27me3 control nucleosomes together to generate a range of histone tail PTM percentages (100% to 1.6%). Similar to the serial dilutions, we found a large degree of linearity and reproducibility for both the H3K4me3 and H3K27me3 (R^2^ ≥ 0.98; coefficient *P* < 0.001) (Fig. 2f and g). Together, these results show that the immuno-qPCR portion of the I-CATCH system has high analytical performance for measuring and normalizing the levels of nucleosomes and histone tail PTMs.

### I-CATCH system measured histone tail PTM levels has high concordance with ChIP-Seq enrichment calls

**Fig. 3:**
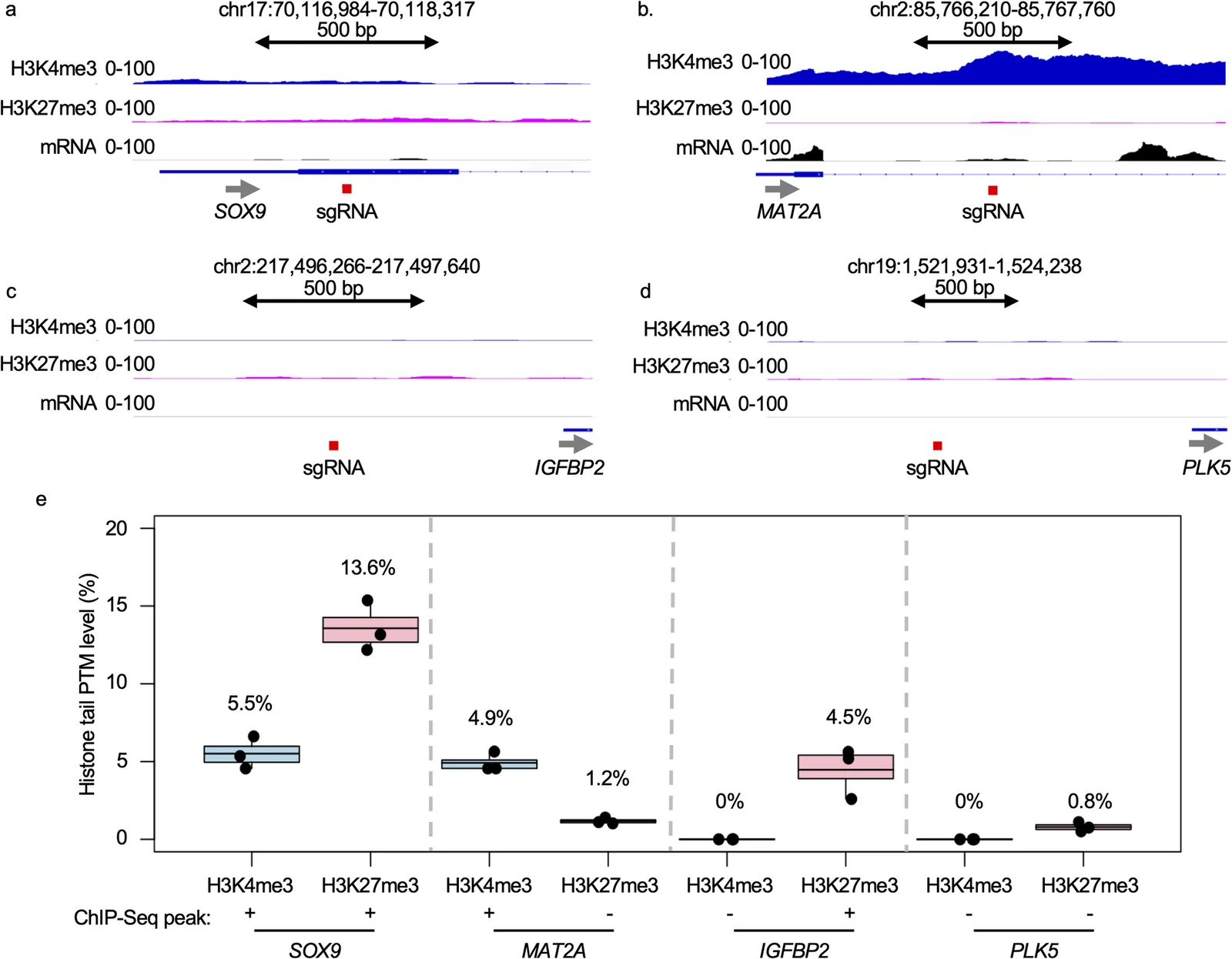
Plots showing ChIP-Seq enrichment levels and RNA-Seq expression levels at genomic loci in proximity to the genes (**a**) SOX9, (**b**) MAT2A, (**c**) IGFBP2, and (**d**) PLK5. (**e**) Boxplot showing levels of histone tail PTMs as determined by the I-CATCH system. *N* = 3 from independent aliquots of each condition. Center line, mean; box limits, upper and lower quartiles; whiskers, 1.5x interquartile range; points, data.

Since we are unaware of another assay that has normalized the levels of nucleosomes with histone tail PTMs at specific genomic loci, we investigated the concordance between ChIP-Seq enrichment calls and the I-CATCH system levels of H3K4me3 and H3K27me3 at multiple genomic loci using chromatin from the well-characterized HeLa cell line. Using ChIP-Seq data from the ENCODE project, we identified 4 genomic loci with different histone tail PTM enrichment patterns, most of which were selected because they are near genes that have been reported to be important for tumor development. These targets included loci in proximity to the genes SOX9, MAT2A, IGFBP2 and PLK5 (Fig. 3a-d). From the Hela cell ChIP-Seq data, the SOX9 loci was enriched for both H3K4me3 and H3K27me3 and have been reported to co-occur^25,26^, the MAT2A loci was enriched for only H3K4me3, the IGFBP2 loci was enriched for only H3K27me3, and the PLK5 loci was not enriched for either H3K4me3 or H3K27me3. The SOX9 and IGFBP2 loci were selected because the levels of H3K4me3 and H3K27me3 have been found to associate with gene expression and has been shown to be important for tumor metastases^27,28^. The MAT2A loci was chosen because it is enriched for H3K4me3 and its expression level has been shown to be important for tumor development^29^. The PLK5 loci was chosen because it was not enriched for either histone tail PTMs and has been found to be expressed in only a few non-proliferative tissues^30^. After determining that the dCas9 no template control had low levels of background signal and that the background signal did not increase in the no dCas9 with HeLa cell chromatin control, we measured the normalized levels of H3K4me3 and H3K27me3 using the I-CATCH system and we found strong concordance between ChIP-Seq enrichment results and I-CATCH levels of each of the targeted loci (Fig. 3e and Supplemental Fig. 1a). However, since the signal for H3K27me3 at PLK5 is below the dCas9 no template control background, the percentage of H3K27me3 may be lower than the calculated 0.8%. The only non-concordant measurement between ChIP-Seq and the I-CATCH system was at the MAT2A loci for H3K27me3. While the ChIP-Seq did not show statistically significant enrichment for H3K27me3 at MAT2A, the I-CATCH system measured low levels (1.2%) of H3K27me3. Although further investigations are required, this result may suggest that the I-CATCH system has greater sensitivity for measuring histone tail PTMs in comparison to ChIP-Seq.

### I-CATCH system can be used to measure histone tail PTM levels from unaltered plasma samples

**Fig. 4:**
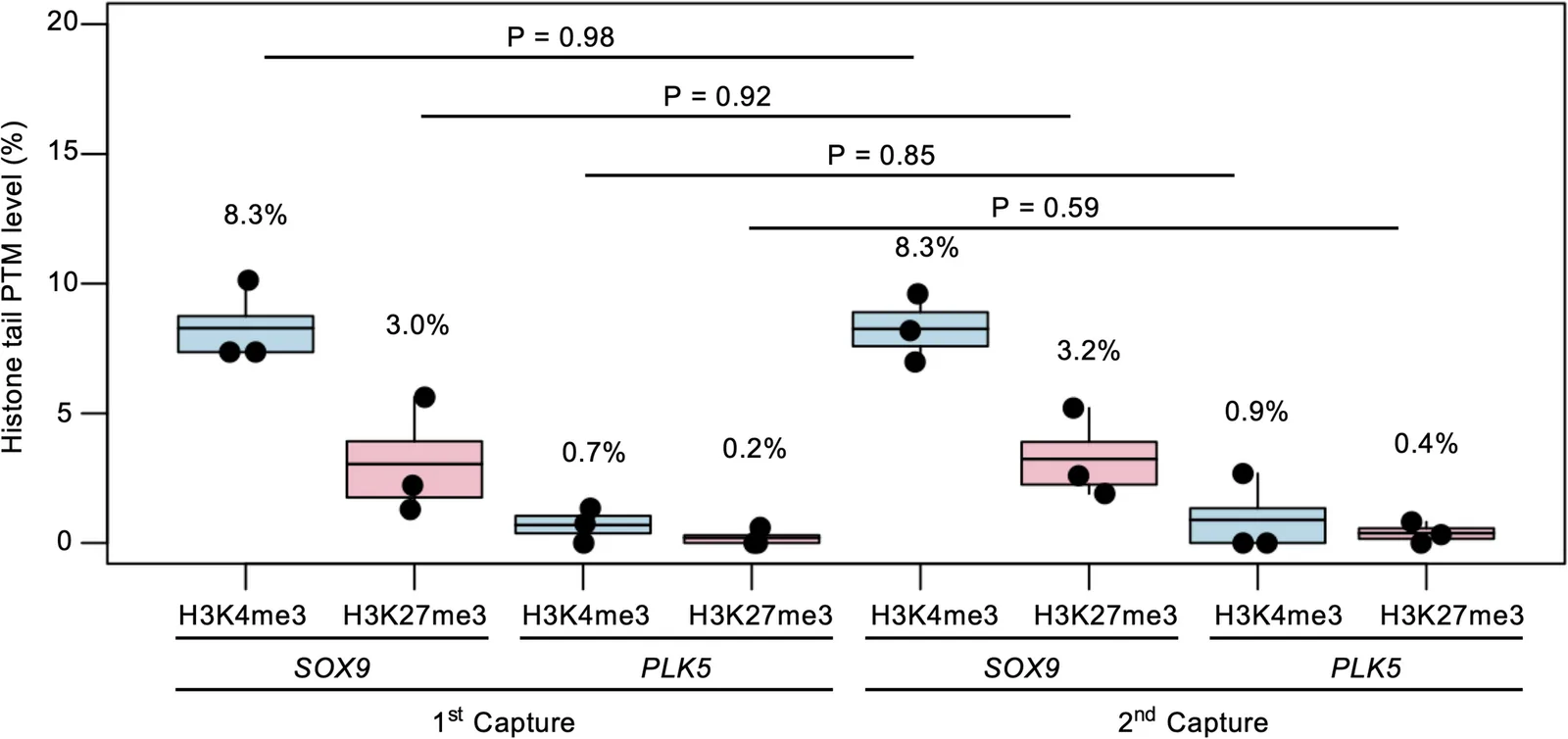
Boxplot showing levels of histone tail PTMs determined by the I-CATCH system using sequential capture of multiple loci from unaltered human plasma. *N* = 3 from independent plasma aliquots of each condition; P = Student’s T-test, two-sided. Center line, mean; box limits, upper and lower quartiles; whiskers, 1.5x interquartile range; points, data.

We then investigated whether the I-CATCH system could be used to measure histone tail PTM levels of cell-free nucleosomes from unaltered human plasma. Since the histone tail PTM levels of the pooled plasma samples from healthy individuals are unknown, we chose to investigate SOX9 because it has a bivalent promoter (enriched for co-occurring H3K4me3 and H3K27me3) and PLK5 because it is likely to have low levels for both histone tail PTMs. Because the current I-CATCH system cannot measure the histone tail PTMs of multiple genomic loci simultaneously, a potential limitation when measuring nucleosomes from samples with limited volume, such as patient plasma, we investigated whether the sequential capture of multiple loci would affect the ability of the I-CATCH system to measure the levels of histone tail PTMs. To investigate the ability of the I-CATCH system to capture nucleosomes from unaltered healthy human plasma and the ability to use sequential capture, we first captured either SOX9 or PLK5 from 100µL of unaltered human plasma using the standard I-CATCH system protocol. After the first loci was captured, we stored the plasma sample in −20˚C overnight. The next day, we thawed the plasma sample and again used the I-CATCH system to measure the histone tail PTM levels of the second loci. Similar to the histone tail PTM levels of HeLa cells, in the plasma samples, we found no evidence of non-specific binding of cell-free nucleosomes to the no dCas9 control and found that SOX9 had positive levels for both H3K4me3 and H3K27me3 and PLK5 had very low levels (< 1%) for both of the histone tail PTMs (Fig. 4 and Supplemental Fig. 1b). Again, similar to the HeLa chromatin result, the H3K27me3 signal at PLK5 was below the background signal of the no template control and the percentages may be lower than the calculated 0.2% and 0.4%. Furthermore, we found that using a sequential capture method to capture multiple loci did not affect the ability of the I-CATCH system to measure the levels of multiple histone tail PTMs. Although we have selected a small number of genomic loci to investigate, our preliminary results provide evidence that the I-CATCH system can measure the levels of multiple histone tail PTMs from unaltered human plasma and that a sequential capture method can be used to capture multiple loci from a single sample.

## Discussion

Here, we introduce the I-CATCH system, a technology designed to measure and normalize the levels of multiple histone tail PTMs at targeted genomic loci. After we found no evidence of antibody steric hindrance, we showed that the immuno-qPCR assay of the I-CATCH system has high analytical performance for measuring and normalizing the levels of nucleosomes and multiple histone tail PTMs. Next, using nucleosomes from HeLa cells, we showed that there was strong concordance between the ChIP-Seq enrichment results and histone tail PTM levels measured using the I-CATCH system. We also showed that the I-CATCH system can measure the levels of nucleosome-bound cell-free DNA and histone tail PTMs from unaltered human plasma samples. Finally, we showed that multiple genetic loci can be captured sequentially using the I-CATCH system. These findings suggest that the I-CATCH system is a promising proof-of-concept methodology to measure and normalize the levels of multiple histone tail PTMs.

While the knowledge regarding DNA methylation alterations far exceeds that of histone tail PTMs, it is likely that the numerous combinatorial patterns of histone tail PTMs encode more epigenetic information in comparison to the binary events of DNA methylation. While the expansion of the multiplexed immuno-qPCR assay in the I-CATCH system to measure more histone tail PTMs is limited by the availability of recombinant nucleosome controls, antibody steric hindrance, and the availability of high-quality antibodies, it is now possible to investigate how the normalized levels of multiple histone tail PTMs interact with each other to ultimately lead to the regulation of gene expression. While in this study we relied on vender-validated ChIP-grade antibodies and a standard curve of recombinant nucleosome controls to quantify and normalize the levels of captured nucleosomes and histone tail PTMs, the captured nucleosomes can also be measured using top-down or middle-down mass spectrometry to determine the accuracy of the immuno-qPCR assay, which would be most informative when validated antibodies are not available or when the PTM signals are near the background level.

Although it is well-established that alterations of histone tail PTMs are important for cancer development, due to technological limitations, the ability to produce clinically relevant information regarding histone tail PTM levels from patient samples has been limited. Here, our preliminary results provide evidence that the I-CATCH system can be used to measure normalized levels of histone tail PTMs from cell-free nucleosomes of plasma samples. This result was not unexpected since we have previously shown that dCas9 can capture nucleosome-bound cell-free DNA from unaltered plasma^21^. Furthermore, the ability to capture specific loci of interest without altering the plasma sample provides a new approach that can be used to expand the amount and type of information from these volume-limited samples.

Although the I-CATCH system overcomes a number of limitations plaguing the field of chromatin biology, it is not without its own limitations. The largest one being that it has low throughput. Since the I-CATCH system targets genomic loci using dCas9, only one locus can be measured at a time. This inherently makes the I-CATCH system ill-suited for discovering new biologically-important alterations. Although an array-based I-CATCH system may be possible, we foresee the I-CATCH system being used in combination with high-throughput ChIP-based technologies, as shown in this manuscript, which is a well-established practice in the field of biomarker discovery. In future studies, we plan to combine the I-CATCH system with new histone tail PTM bioinformatic discovery tools to investigate the clinical utility of histone tail PTM biomarkers.

Another limitation of the I-CATCH system is that it cannot distinguish whether histone tail PTMs co-exist or co-occur. However, we believe that the immune-qPCR proximity extension assay, which is a technology designed to determine when antibodies bind in close proximity^31,32^, could also be incorporated into the I-CATCH system to help distinguish between co-existing and co-occurring histone tail PTMs. Another strategy would be to incorporate the I-CATCH system into a digitized independent immuno-qPCR reaction, which also has the benefit of increasing the sensitivity and multiplexing potential^33^. However, the limitation of digitizing the I-CATCH system is that it is unable to determine whether co-occurring histone tail PTMs occur on the same or different tails of the nucleosome.

In conclusion, with the I-CATCH system, the normalized levels of multiple histone tail PTMs at targeted genomic loci can be determined. In future studies, we plan to investigate how the normalized levels of multiple histone tail PTMs interact to regulate the expression of genes important for tumor progression and whether these alterations can be used as clinical biomarkers.

## Methods

### Antibody conjugation

The antibodies used in this study was anti-histone3 (Histone H3 Recombinant Rabbit Monoclonal Antibody (24HC2 LC12), ThermoFisher, Catalog 701517, lot 2247099, Recombinant Monoclonal [24HC2 LC12], Reactivity: Human), anti-H3K4me3 (H3K4me3 Antibody, SNAP-Certified™ for CUT&RUN and CUT&Tag, EpiCypher, Catalog 13–0060, lot 24008001-82, Monocolonal [2909-3D7], Reactivity: Human), and anti-H3K27me3 (H3K27me3 Antibody, SNAP-Certified™ for CUT&RUN and CUT&Tag, EpiCypher, Catalog 13–0055, lot 23072002-91, Monoclonal [2084-1G5], Relativity: human). All were vendor-validated as ChIP-grade.

To conjugate the barcode DNA sequences (Table 1) to the antibodies, anti-histone3, anti-H3K4me3, and anti-H3K27me3, we first equilibrated 14 µl (1 mg/ml) of the antibodies with phosphate buffer (100 mM phosphate buffer; pH 7.3) using a 75 µl 40 kDa Zeba column (Zeba 87765). The equilibrated antibodies were activated by adding 2 µL of 1.452 mg/mL Sulfo-SMCCC (Thermo Fisher 22322) and incubated at 4˚C for 2 hr. Next, the treated antibodies were equilibrated with phosphate EDTA buffer (100 mM phosphate buffer; pH 7.3 + 20 mM EDTA) using a 0.5 mL 40 kDA Zeba column (Zeba 87767). The 5’-thiol modified oligos (IDT) were resuspended to 500 µM in phosphate EDTA buffer. Next, 2.6 µL oligos were reduced by adding 4.4 µL 40 mM DTT (Thermo Fisher R0861) and incubated at 95˚C for 2 min then 37˚C for 1 h. The reduced oligos were then equilibrated with phosphate buffer using two 0.5 mL 7 kDA Zeba columns (Zeba 89883). The antibody and oligo concentrations were measured using a Nanodrop. The SMCC-treated antibodies were mixed with DTT treated oligo at 1 Ab:10 Oligo molar and incubated overnight at 4˚C. The antibody-oligo conjugates were then purified and equilibrated using phosphate buffer with a 0.5 mL 40 kDa Zeba column. The oligo-antibody conjugation was confirmed using poly-acrylamide gel electrophoresis, and the concentration of the purified antibody conjugates were measured using the Pierce BCA Protein Assay Kit (Thermo Scientific PI23227).

### Bead blocking

For each reaction, we used 0.125 µl of streptavidin beads (Dynabeads -Streptavidin M280), which were first washed using 50 µl of PBS 3 times before being blocked in 50 µl of Blocking buffer (10 mM Tris with 150 mM NaCl and 1% (w/v) BSA (NEB B9000S) for 30 min at room temperature in a mini tube rotator (Fisher Scientific 260750 F) set at the highest speed. After 30 min, the beads were placed on a magnet, the Blocking buffer was removed, and the beads were resuspended using Binding buffer (10 mM Tris with 150 mM NaCl and 0.5% (w/v) BSA), nucleosome mixture or human plasma.

### Recombinant mononucleosome controls

The recombinant biotin-tagged mononucleosomes were purchased from EpiCypher (H3K4me3 16–0316-20), (H3K27me3 16–0317-20) and (H3K4me3, H3K27me3 16–0403). To investigate the steric hindrance and the analytical performance of the immuno-qPCR, a total of 1 µl of 15.6 pg/ml of mononucleosomes control was added to 19 µl of Binding buffer and was used to resuspend the blocked beads. After the mixture was incubated in a mini tube rotator for 1 h at room temperature, the nucleosome bead complexes were captured using a magnet and were washed 3 times using dCas9 Washing buffer (10 mM Tris; pH 8.0, 150 mM NaCl, and 0.05% tween 20). The captured nucleosomes were then resuspended and blocked in 20 µl of Blocking buffer for 30 min. The blocked captured mononucleosomes were then washed 3 times with dCas9 washing buffer and blocked again with 20 µl of StartingBlock™ (ThermoScientfic 37578) for 5 min.

### Tagging the nucleosome with the antibody conjugates

After blocking the nucleosomes with StartingBlock™, 20 µl of Antibody Binding buffer (1X PBS with 1% BSA, 100 µg/mL goat serum 0.05% Tween-20) with the conjugated antibody (1 µl 100 pM anti-histone 3, 1 µl 10 pM anti-H3K4me3 and/or 1 µl 100 pM anti-H3K27me3) was added to the captured nucleosomes and incubated at room temperature for 15 min on the tube rotator. The antibody-tagged nucleosomes were then washed 3 times with dCas9 Washing buffer before washing 2 times with PBS. The tagged nucleosomes were then eluted with 2 µl of H_2_0 and added to the PCR reaction.

### Nested barcoded qPCR

The nested PCR assay procedure consists of two sequential PCR reactions. In Step 1 PCR, pre-amplification, 2 µl of the captured substrate was added to 18 µl of reaction buffer [200 µM deoxynucleotide triphosphates, 16.6 mM (NH_4_)_2_SO_4_, 67 mM Tris (pH 8.8), 6.7 mM MgCl_2_, 10 mM β-mercaptoethanol, 0.04 unit of Platinum Taq (Invitrogen 15966005) and 300 nM each of the forward and reverse primers] (Table 1). Conditions used for this PCR step were: 95 °C for 5 min, followed by 10 cycles of 95 °C for 30 s, 56 °C for 45 s, and 72 °C for 45 s, with a final extension cycle of 72 °C for 7 min. The reaction was carried out in a Bio-rad C1000 Touch Thermal Cycler (Bio-rad). The PCR products were diluted to 100 µl with H_2_O, placed on a magnet stand and the amplicons in the aqueous solution were recovered and stored at 4 °C.

For Step 2 PCR (Quantitative multiplexed PCR of amplicons), 2 µl of the diluted amplicon DNA was added to 23 µl qPCR reaction buffer [16.6 mM (NH4)2SO4, 67 mM Tris (pH 8.8), 6.7 mM MgCl_2_, 10.0 mM β-mercaptoethanol, 200 µM deoxynucleotide triphosphates, 0.04 units Immolase DNA polymerase (bioline 21046), 300 nM each of primers (forward and reverse) and 100 nM labeled probe (IDT) (Table 1). The reaction was carried out in a Bio-rad CFX96 Real-Time System (Bio-rad). The reaction conditions were: 95 °C for 10 min, followed by 40 cycles of 95 °C for 15 s and 65 °C for 1 min.

### Measuring the levels of histone tail PTMs in HeLa cells and human plasma

#### Assembling RNPs

For each reaction, 2 ul of 170 pg/ml dCas9-3XFLAG™ Biotin Protein (MilliporeSigma DCAS9PROT) was incubated with 2 µl of 10 fM sgRNA (IDT Table 1) for 15 min at room temperature in a mini tube rotator.

#### Cell line and plasma chromatin

Purified HeLa Polynucleosomes was purchased from Epicypher (16 − 0003) and 4 µl of 1 µg/ml of polynuclesomes were added to 92 µl of Binding buffer and mixed with the RNPs. Human plasma was purchased from BioIVT (K_2_EDTA plasma, BioIVT, Hicksville, NY) and consisted of pooled plasma from gender-unspecific healthy individuals. For each reaction, 96 µl of plasma was used per reaction.

After incubating the RNPs with the HeLa nucleosomes or plasma for 3 h, the RNPs were captured using blocked streptavidin beads. After 15 min, the bead-RNP nucleosomes complexes were pulled down using a magnet. The unaltered plasma was removed and stored at −30˚C between 1 and 3 days before being used again to investigate sequential genomic loci capture. The captured nucleosomes were then washed, blocked, and tagged with the conjugated antibodies using the steps described above.

#### ChIP-Seq and RNA-Seq analysis

Hela cell ChIP-Seq raw data from the ENCODE project was downloaded from the UCSC database^34^. ChIP-Seq data was aligned to hg19 genome using BWA and enriched peaks were called using macs3 using the p-value cutoff of 0.01^35,36^. The HOMER software tool annotatePeaks.pl was used to annotate the enriched peaks^37^. To construct the sgRNA, we selected 300 nt up and downstream of the enriched peaks and input the sequences into the IDT custom guide RNA designer. The sgRNA with the best combined sensitivity and specify score was chosen. The Hela Cell RNA-Seq raw data from the ENCODE project was downloaded from the UCSC database^34^ and was aligned to hg19 using Hisat2^38,39^. The software IGV was used to visualize the RNA-Seq and ChIP-Seq data^40^.

#### Statistical measurements

The R function t.test (two-sided) was used to calculate the P-values when comparing Ct and histone tail PTM levels in the steric hinderance and sequential capture experiments. The R function lm was used to calculate the slopes and the function coef was used to calculate the slopes coefficient P-values when measuring the performance of the immuno-qPCR to measure a range of nucleosome concentrations and range of histone tail PTM percentages.

#### Statistics & reproducibility

No statistical method was used to predetermine sample size. No data were excluded from the analyses. We did not use randomization in this study. The investigators were not blinded to allocation during experiments and outcome assessment.

## Supplementary Information

Below is the link to the electronic supplementary material.

Supplementary Material 1

## Data Availability

All data supporting the findings of this study are available within the paper and its Supplementary Information. Hela cell ChIP-Seq and RNA-Seq raw data from the ENCODE project was downloaded from the UCSC database https://genome.ucsc.edu.

## Code availability

The custom code used for the analyses in this study is available upon reasonable request. R version 4.2.3 was used for the analysis in this manuscript. Reprints and permissions information is available at www.nature.com/reprints.
