# Supplementary material for: Defining the levels of the chromatin landscape using the I-CATCH system
Source: Sci Rep. 2026 Aug 6;16:34590. doi: 10.1038/s41598-026-65001-w (PMC13713579; doi:10.1038/s41598-026-65001-w)
Supplement: Supplementary file 1 [file 41598_2026_65001_MOESM1_ESM.pdf]

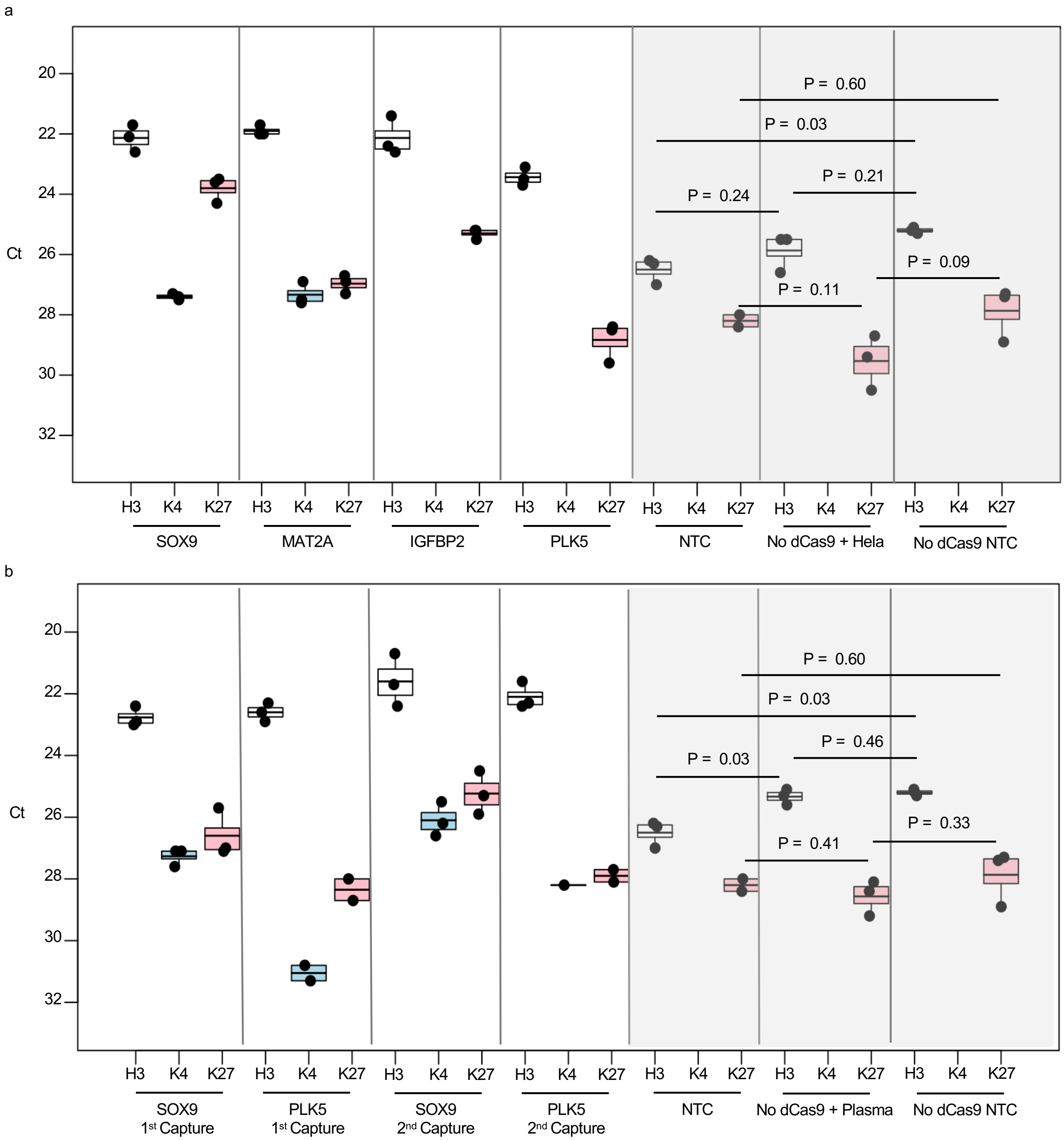


**Supplemental Figure 1.** Box plot shows the Ct values of the I-CATCH system measuring different loci in **a** HeLa cells and **b** plasma. N=3 from independent aliquots of each condition; Missing datapoints are due to non-amplified targets. P = Student's T-test, two-sided; NTC = no template control; Center line, mean; box limits, upper and lower quartiles; whiskers, 1.5x interquartile range; points, data.
